# Conditional survival of metastatic renal cell carcinoma patients treated with high-dose interleukin-2

**DOI:** 10.3332/ecancer.2016.676

**Published:** 2016-09-29

**Authors:** David M Gill, David D Stenehjem, Kinjal Parikh, Joseph Merriman, Arun Sendilnathan, Archana M Agarwal, Andrew W Hahn, Sumati Gupta, Srinivas Kiran Tantravahi, Wolfram E Samlowski, Neeraj Agarwal

**Affiliations:** 1 University of Utah Huntsman Cancer Institute, Salt Lake City, UT 84112, USA; 2 Department of Pharmacotherapy, College of Pharmacy, University of Utah, Salt Lake City, UT 84112, USA; 3 Department of Genitourinary Medical Oncology, The University of Texas MD Anderson Cancer Center, Houston, TX 77030, USA; 4 Department of Internal Medicine, Vanderbilt University, Nashville, TN 37232, USA; 5 University of Cincinnati Medical Center, Cincinnati, OH 45219, USA; 6 Department of Pathology and ARUP Laboratories, The University of Utah, Salt Lake City, UT 84108, USA; 7 Comprehensive Cancer Centers of Nevada, Las Vegas, NV 89148, USA; † Equal contribution

**Keywords:** metastatic renal cell cancer, high-dose interleukin-2, conditional survival

## Abstract

Conditional survival (CS) is a clinically useful prediction measure which adjusts a patient’s prognosis based on their duration of survival since initiation of therapy. CS has been described in numerous malignancies, and recently described in patients with metastatic renal cell carcinoma (mRCC) who received vascular endothelial growth factor tyrosine kinase inhibitor (VEGFTKI) therapy. However, CS has been not reported in the context of mRCC treated with high-dose interleukin-2 therapy (HDIL-2). A total of 176 patients with histologically confirmed metastatic clear cell RCC (mccRCC) treated with HDIL-2 at the University of Utah Huntsman Cancer Institute from 1988–2012 were evaluated. Using the Heng/IMDC model, they were stratified by performance status and prognostic risk groups. Two-year CS was defined as the probability of surviving an additional two years from initiation of HDIL-2 to 18 months after the start of HDIL-2 at three-month intervals. The median overall survival (OS) was 19.9 months. Stratifying patients into favourable (n = 35; 20%), intermediate (n = 110; 63%), and poor (n = 31; 18%) prognostic groups resulted in median OS of 47.5 (HR 0.57, 95% CI 0.35–0.88, p = 0.0106 versus intermediate), 19.6 (HR 0.33, 95% CI 0.10–0.33, p < 0.0001 versus poor), and 8.8 (HR 5.34, 95% CI 3.00–9.62, p < 0.0001 versus favourable) months respectively. Two-year overall CS increased from 43% at therapy initiation to 100% at 18 months. These results have significant ramifications in prognostication. Furthermore, it is important when counseling patients with mccRCC who have completed treatment with HDIL-2 and are in active follow-up.

## Introduction

Renal cell carcinoma (RCC) is the most common primary renal neoplasm, accounting for over 80% of cases with incidence being 60% more common in men. There are greater than 60,000 new cases and 14,000 deaths attributed to RCC in the United States annually [1]. As a subtype of RCC, clear cell (ccRCC) accounts for the vast majority of cases [2].

HDIL-2 was approved for treatment of patients with mRCC in 1992. Approval was based in durable responses seen in a subset of patients (~10%) treated with HDIL-2 [3]. Recent studies of HDIL-2 have shown an overall response rate of up to 25% [4]. Furthermore, large retrospective studies recently reported an additional ~30% who do not experience an objective response, but stable disease as the best response [5]. Survival outcomes in these patients achieving stable disease as best response were similar to those achieving partial response as the best response [5–6]. HDIL-2 was the mainstay of therapy for mRCC until 2005, when large randomised trials revealed that targeted therapies consisting of vascular endothelial growth factor tyrosine kinase inhibitors (VEGFTKIs) and mammalian target of rapamycin (mTOR) inhibitors had improvements in progression free survival (PFS), ORR, and OS. However, because of the long and durable responses seen in a subset of patients, current National comprehensive cancer network (NCCN) guidelines continue to recommend HDIL-2 for patients with good performance status and intact organ function. With the emergence of checkpoint inhibition (nivolumab), there has been a resurgence of cancer immunotherapy for mRCC, and an improved appreciation of benefits of immunotherapy in general among oncology providers [7].

Multiple prognostication tools are available to help stratify an individual patient’s risk and help guide therapy. These include the International Metastatic RCC Database Consortium (IMDC) or Heng model [8], Memorial Sloan-Kettering Cancer Center (MSKCC) or Motzer model [9], Cleveland Clinic Foundation model [10], French Model [11], and the International Kidney Cancer Working Group (IKCWG) model [12]. Each model utilises a combination of clinical factors such as performance status (European Cooperative Oncology Group (ECOG) or Karnofsky), time from diagnosis to treatment, previous treatments, basic laboratory values, and metastatic lesions. These models vary somewhat, but have been externally validated and are fairly concordant. Risk stratifying patients into separate groups reveals the great heterogeneity in disease prognosis based on these risk factors. A 2011 article using the IMDC model revealed patients in the favourable risk group had median OS of 43.2 months compared to 7.8 months for patients in the poor risk group. Rate of death for patients in the favourable versus poor risk group was 0.30 and 0.88 respectively [8].

These projections are crucial to patient education, counseling, prognostication, and therapeutic decisions. However, when a patient outlives their projected survival, it can be difficult to provide accurate prognostic information. An adjustment to a patient’s prognosis on the basis of survival since treatment initiation or therapy duration is termed conditional survival (CS). In the same manner that the above risk models provide valuable clinical information, CS is vital for patients who surpass anticipated median survival. CS has been reported in the setting of treatment of many malignancies, including in those patients with mRCC treated with VEGFTKIs [13]. However, this is the first study examining CS in the context of mRCC patients treated with HDIL-2. Improved understanding of CS in this setting has the potential to have important implications on counseling for these patients, and plan for follow up after completion of therapy with HDIL-2.

## Methods

Patients with histologically confirmed clear cell mRCC treated with HDIL-2 at the University of Utah Huntsman Cancer Institute were evaluated. Data was collected on patients treated from 1988–2012 using a uniform template. Information was gathered from the patient’s medical record or publically accessible information. All sequential patients with clear cell mRCC treated with at least one dose of HDIL-2 were identified. Patients were excluded if date of last follow-up or death was not available, or date of HDIL-2 administration was not recorded. Local institutional review board (IRB) approval was obtained prior to data collection. Patients of all performance statuses and risk groups were included in analysis. The International metastatic renal cell carcinoma database consortium (IMDC) model was used to prognosticate patient outcomes. Specifically measuring two-year CS in this study, we measured the probability of surviving an additional two years at each three-month interval after initiation of HDIL-2 estimated by Kaplan-Meier methodology as determined by IMDC risk model. These time points were chosen prior to study initiation and were performed in a similar analysis to the Harshman *et al* study [13]. Once a patient died or did not have further follow-up, he was excluded from further CS calculations at subsequent three month intervals. For example, a patient who died 13 months from HDIL-2 initiation would be included in each CS calculation prior to the 15 month landmark but would be censored from subsequent (15 and 18 month) calculations. Analysis was stratified by all patients and stratified by risk score (favourable, intermediate, and poor).

## Results

**Figure 1.**
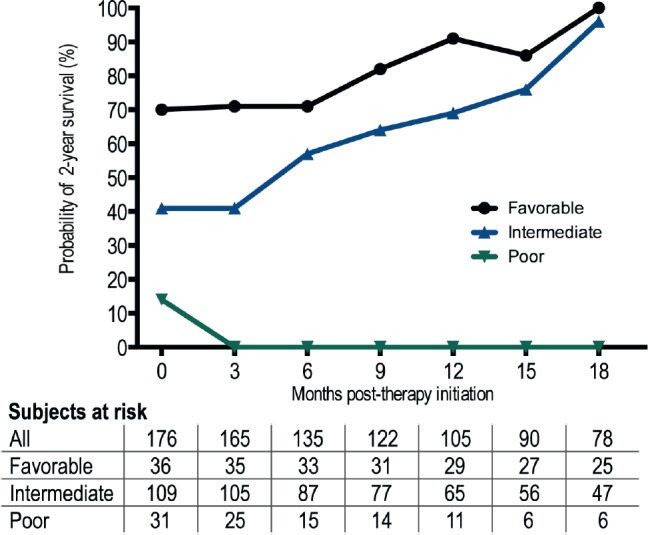
Probability of surviving two additional years in patients receiving HDIL-2 by Heng/IMDC Criteria.

A total of 176 evaluable patients who were eligible were included with a median age of 55 years (range 13–76 years) and 80% (n = 140) were male. Baseline patient characteristics and clinical outcomes on treatment with HDIL-2 including response rates, and survival outcomes are described in Table 1. Median OS from HDIL-2 administration was 19.9 months. Stratification by Heng/IMDC criteria at therapy initiation resulted in OS estimates for the favourable (n = 35; 20%), intermediate (n = 110; 63%), and poor (n = 31; 18%) prognostic groups of 47.5 (HR 0.57, 95% confidence interval (CI) 0.35–0.88, p = 0.0106 versus intermediate), 19.6 (Hazard ratio (HR) 0.33, 95% CI 0.10–0.33, p < 0.0001 versus poor), and 8.8 (HR 5.34, 95% CI 3.00–9.62, p < 0.0001 versus favourable) months respectively. Two-year overall CS increased from 43% at therapy initiation to 100% at 18 months (Table 2). After three months in the poor prognosis arm, two-year CS was not reached by any patient. Two-year CS stratified by favourable, intermediate, and poor prognostic risk groups is presented in Table 2 and Figure 1.

## Discussion

Although several novel therapies have been approved over the last decade for the treatment of mRCC, including VEGF-TKIs, mTOR inhibitors, and more recently an immune checkpoint inhibitor (nivolumab) and a c-MET inhibitor (cabozantinib), the median OS of these patients is still less than three years [7, 14–15]. HDIL-2 is the only therapy which has consistently been shown to be associated with long term remissions in a subset of these patients. Accordingly, current NCCN guidelines continue to recommend HDIL-2 for patients with good performance status and intact organ function, and HDIL-2 continues to play a significant role in the treatment of these patients. Although variety of risk models exist to help stratify patients depending on their risk and survival studies extrapolated from clinical trials exist, there is no dynamic tool for assessing survival probability in mRCC patients treated with HDIL-2. Although several pre-treatment prognostic nomograms have been reported, none apply to those patients who have completed treatment with HDIL-2 and are in active follow-up. Our results illustrate that for patients with mRCC treated with HDIL-2 and Heng/IMDC model intermediate and favourable risk, the overall two-year survival increases steadily as a patient reaches each three-month survival landmark after therapy initiation. For example, there is a 50% increase in overall two-year survival for a patient in the intermediate risk group who survives nine months from therapy initiation (64% versus 41%). For favourable risk patients, there is a 91% overall two-year survival once they have survived 12 months from therapy initiation. These results have important implications for patient care in this setting, i.e. with regard to counseling for these patients, clinical decision making concerning interval of follow-up scans, and regarding subsequent systemic therapies. These data points are also designed to be easily applicable to the clinic setting as they correlate with common follow-up imaging intervals.

With any retrospective dataset, there are limitations regarding those unintentionally excluded from analysis. We sought to minimise this with thorough data review of over 24 years of patient data and broad inclusion data. Though this study provides prognostic projections for a specific subset of patients, i.e. those with mRCC treated with HDIL-2 in a single institution with adequate follow-up, it is still somewhat limited by sample size relative to other CS studies. Future studies are needed to study CS after two years and may require multiple academic institutions given the relatively small number of patients receiving HDIL-2. With any single-institution study, external validation should be considered prior to implementation of CS into the clinical practice.

## Conclusion

To conclude, CS in the context of HDIL-2 therapy in the mRCC setting has the potential to become a clinically useful prediction measure that adjusts prognosis of these patients on the basis of time elapsed since HDIL-2 treatment initiation. It may have important implications in the counseling for these patients and also clinical decision making after completion of HDIL-2 treatment.

## Competing interests

All authors have no financial or non-financial competing interests to disclose. No sources funded this research.

## Figures and Tables

**Table 1.** Baseline characteristics and clinical outcomes.

Demographic/clinical characteristic	n = 176
Median age (range)	55 (13–76)
Males (%)	140 (79%)
IMDC criteria (n, %)	
Favourable	36 (20%)
Intermediate	109 (62%)
Poor	31 (18%)
Best Response to HDIL-2	
CR	16 (9%)
PR	11 (6%)
Stable disease (SD)	52 (30%)
Progressive disease (PD)/Not evaluable (NE)	97 (58%)
PFS (median)	5.6
OS (median)	19.9

**Table 2.** Two-year CS of patients with mRCC treated with HDIL-2.

Months from HDIL-2 initiation	Two-yr overall CS at time points	Two-yr CS stratified by Heng/IMDC risk groups		
		Favourable	Intermediate	Poor
0	43% (176)	70% (36)	41% (109)	14% (31)
3	44% (165)	71% (35)	41% (105)	0% (25)
6	61%(135)	71% (33)	57% (87)	0% (15)
9	65% (122)	82% (31)	64% (77)	0% (14)
12	75% (105)	91% (29)	69% (65)	0% (11)
15	83% (90)	86% (27)	76% (56)	0% (6)
18	100% (78)	100% (25)	96% (47)	0% (6)
